# Impact of SARS-CoV-2 Infection on Erythropoietin Resistance Index in Hemodialysis Patients

**DOI:** 10.3390/geriatrics10020033

**Published:** 2025-02-24

**Authors:** Guido Gembillo, Luca Soraci, Luigi Peritore, Rossella Siligato, Vincenzo Labbozzetta, Alfio Edoardo Giuffrida, Felicia Cuzzola, Claudia Spinella, Adolfo Romeo, Vincenzo Calabrese, Alberto Montesanto, Andrea Corsonello, Domenico Santoro

**Affiliations:** 1 Unit of Nephrology and Dialysis, Department of Clinical and Experimental Medicine, University of Messina, 98122 Messina, Italy; luigiperitore1994@gmail.com (L.P.); vincenzo.labbozzetta@gmail.com (V.L.); alfiogiuffrida91@libero.it (A.E.G.); feliciacuzzola@gmail.com (F.C.); spinella.claudia@gmail.com (C.S.); romeo.adolfo@alice.it (A.R.); v.calabrese@outlook.it (V.C.); 2 Unit of Geriatric Medicine, Italian National Research Center on Aging (IRCCS INRCA), 87100 Cosenza, Italy; l.soraci@inrca.it (L.S.); a.corsonello@inrca.it (A.C.); 3 Nephrology Unit, University Hospital of Ferrara, 44121 Ferrara, Italy; rossellasiligato@gmail.com; 4 Department of Biology, Ecology and Earth Sciences, University of Calabria, 87036 Arcavacata di Rende, Italy; alberto.montesanto@unical.it; 5 Department of Pharmacy, Health and Nutritional Sciences, University of Calabria, 87036 Rende, Italy

**Keywords:** Sars-CoV-2, COVID-19, hemodialysis, renal replacement therapies, anemia, kidney failure, erythropoietin, chronic kidney disease

## Abstract

**Background/Objectives:** Hemodialysis (HD) patients with advanced chronic kidney disease (CKD) are highly vulnerable to complications from SARS-CoV-2 infection. Anemia management in this population is complex, particularly due to erythropoietin resistance, which may be exacerbated by COVID-19-related inflammation. To this aim, in this small-scale retrospective study, we investigated trends in the erythropoietin resistance index (ERI) over time in patients with and without SARS-CoV-2 infection. **Methods:** This single-center retrospective study included 25 HD patients, divided into two groups: 15 with a history of SARS-CoV-2 infection (CoV2 group) and 10 without (nonCoV2 group). The ERI was assessed over four visits, with 70–100-day intervals between them. Linear mixed models were used to evaluate factors associated with ERI changes. **Results:** Patients in the CoV2 group exhibited significantly higher ERI increases between T1 (baseline) and T2 (post-infection) compared to the nonCoV2 group (median ΔERI: +4.65 vs. −0.27, *p* < 0.001). During the T2–T4 recovery period, CoV2 patients demonstrated a delayed but substantial decline in the ERI, converging to baseline levels by T4. Male sex and hemoglobin levels were negatively associated with the ERI. **Conclusions:** SARS-CoV-2 infection induces transient but significant erythropoietin resistance in HD patients, likely due to inflammation and disrupted erythropoiesis. Tailored anemia management strategies, including the potential use of hypoxia-inducible factor stabilizers, are warranted. Larger, multicenter studies are needed to validate these findings and improve treatment protocols.

## 1. Introduction

Chronic kidney disease (CKD) is a progressive disease characterized by a deterioration in kidney function over time, and represents a relevant public health problem worldwide [1,2]; indeed, it has a great impact on patients’ morbidity, mortality, and healthcare costs, becoming one of the leading causes of death and disability of the present day [3,4,5].

SARS-CoV-2 infection has recently emerged as a dangerous contributor to the progression of kidney dysfunction in patients with CKD, usually because of systemic immunological activation and/or ischemic injury. Among patients with advanced CKD, those undergoing RRT, such as hemodialysis (HD), are particularly vulnerable to severe COVID-19 outcomes due to their compromised immune status, due to both uremia and chronic inflammation, as well as a higher prevalence of comorbidities [6,7,8].

One of the main complications of advanced CKD that has an adverse impact on individual patient outcomes is represented by anemia, whose onset is mainly determined by a decreased production of endogenous erythropoietin (EPO) and impaired iron availability with functional deficiency [9]; as a consequence, the administration of exogenous iron and EPO treatment represent the mainstays for the management of this complication [10,11], despite not always prescribed to hospitalized patients [12]. The management of chronic anemia in patients with advanced CKD may become particularly complex when superimposed with SARS-CoV-2 infection; indeed, the acute inflammatory response enhanced by viral infection can exacerbate functional iron deficiency and the inadequate utilization of iron stores, decrease iron absorption through enhanced hepcidin production, suppress EPO production, direct viral bone marrow invasion, and activate autoimmune cascades against red blood cells [13,14,15]. The presence of anemia in patients with COVID-19 has been associated with poor prognosis; in a recent meta-analysis including 57,563 patients with COVID-19, lower hemoglobin levels were associated with more severe disease courses and worse outcomes [16,17,18]. Moreover, hemoglobin (Hb) levels have been used for the risk stratification of hospitalized patients with COVID-19 [19]. Despite SARS-CoV-2 infection being associated with a suppressed production of endogenous EPO [15] and resistance, the administration of human recombinant EPO to COVID-19 patients with anemia requires a careful assessment of the benefits of maintaining adequate hemoglobin levels against the risks of thrombotic events, particularly in the pro-inflammatory and pro-thrombotic state induced by COVID-19. In this context, evidence from the current literature leads to contrasting results. On one hand, some studies have indeed shown that lower serum EPO levels were associated with more severe COVID-19 and poor prognosis [20,21,22]; on the other hand, other ones show a very limited efficacy of the treatment [23,24]. However, viral infection can dramatically increase the resistance to exogenous EPO administration, as already shown during other inflammatory conditions [25,26,27]; for this reason, the evaluation of the erythropoietin resistance index (ERI) should be considered in weighing risks and benefits of EPO administration to COVID-19 patients. In this regard, in the present small-scale retrospective study, we evaluated the impact of SARS-CoV-2 infection on ERI trends over time in a small group of patients with advanced CKD undergoing maintenance HD.

## 2. Materials and Methods

### 2.1. Study Population

This single-center retrospective study was performed in patients treated with subcutaneous erythropoietin and receiving maintenance HD between October 2020 and January 2023 at the Unit of Nephrology and Dialysis of the University Hospital “G. Martino” in Messina, Italy. The inclusion criteria were as follows: age over eighteen years, HD therapy longer than three months and in stable condition, use of an arteriovenous fistula, and anuria. The exclusion criteria were as follows: (1) severe cardiac or cerebral disease, (2) infections that occurred within the last month, (3) active liver disease or cancer, (4) recent surgical procedures or blood transfusions, and (5) pure red cell aplasia or active bleeding.

All included patients (*n* = 25) received HD three times a week for four hours each using heparin anticoagulants. Dialysate flow was 500 mL/min and blood flow was 250–300 mL/min. The dialysate consisted of the following chemicals: calcium 1.50 mmol/L, potassium 2.5 mmol/L, and sodium 140 mmol/L. The presence of SARS-CoV-2 infection was periodically evaluated by conducting Real-Time PCR (RT-PCR) analyses on nasopharyngeal swabs at the time of the study visits. Patients were retrospectively divided into 2 groups: 15 patients with a history of SARS-CoV-2 infection (CoV2 group) and 10 patients without SARS-CoV-2 history (nonCoV2 group). For each patient, a total of 4 visits at regular time intervals of 70–100 days were retrospectively evaluated and included in the study. For patients in the CoV2 group, the baseline visit was considered as the most recent visit before the ascertainment of SARS-CoV-2 infection. Patients were all negative for SARS-CoV-2 at the baseline visit (T1); CoV2 patients were positive for SARS-CoV2 at T2 in contrast to nonCoV2 ones. The research protocol was approved by the Local Ethics Committee for Medical Research of Messina (C.E. prot. n. 03-22, approved on 15th March 2022) and carried out in accordance with the 1964 Declaration of Helsinki and its later amendments.

### 2.2. ERI

All patients underwent a comprehensive assessment that included age, sex, weight measurement, comorbidities documented using ICD-9-CM diagnosis codes (hypertension, cancer, diabetes mellitus, and cerebrovascular disease), and laboratory parameters such as hemoglobin, ferritin, transferrin, transferrin saturation, creatinine, and electrolytes. Medications taken by the patients were also assessed and categorized using Anatomical Therapeutic Chemical (ATC) classification codes to ensure the standardized documentation of drug usage.

The erythropoietin resistance index (ERI) was calculated at each time point as the average weekly erythropoietin (EPO) dose per kg body weight (wt) per average Hb over 3 months (ERI = (EPO/wt)/Hb).

### 2.3. Statistical Analysis

To compare the continuous variables between the 2 groups, an independent samples t-test was used in cases of normality and the Mann–Whitney U test was used otherwise. Continuous variables with a normal distribution were represented by age and length of dialysis; conversely, laboratory values and the ERI were not normally distributed. For categorical variables, Fisher’s exact test was used because of low numbers. The distribution of study variables was evaluated with the Kolmogorov–Smirnov test.

Comparisons between the ERI values across the 4 time points were evaluated using the Mann–Whitney U test (for sub-analysis between two time points) and Friedman’s test for repeated measures. Comparisons between the median ERI across groups and time points were graphically displayed. To compare the evolution over time of the ERI between the nonCoV2 and CoV2 groups, a family of linear mixed models was fitted, adopting time as a continuous variable, group (nonCoV2 and CoV2) and the interaction term between time and group as fixed effects, and subject as a random effect. In order to improve the model’s capacity to capture the two distinct directions of continuous ERI change over time (increase vs. decrease), we fitted two distinct linear mixed models: one including time points from the baseline visit (T1) to the second one (T2); the second including time points from T2 to the fourth (T4) one. Additional models were fitted using time as a categorical variable.

As there was a significant interaction between time and group, we looked at the difference in median ERI per measured time point between the 2 groups. *p* values were adjusted for multiple testing using Holm correction. The goodness of fit of the obtained models was based on the evaluation of the Bayesian Information Criterion and R2. Statistical analysis was performed using R 3.5.2 (R Foundation for Statistical Computing, Vienna, AT, Austria, 2018). Statistical significance was set at a 2-tailed *p*-value < 0.05.

## 3. Results

### 3.1. Descriptive Characteristics of the Study Population

Clinical and demographic characteristics of the study population and the two groups are reported in Table 1. The 25 patients included in the study had a mean age of 66.5 ± 14.9 years and were mostly men (62.5%); the most common comorbidities were hypertension (88%), followed by cerebrovascular disease and diabetes. No significant differences in age, sex, or comorbidities were found between the patients with and without SARS-CoV-2 infection, but a nonsignificant higher prevalence of hypertension, diabetes, and cerebrovascular disease characterized the CoV2 group compared to the nonCoV2 one. Similar to the clinical data, the laboratory values measured at the baseline visit were not significantly different between the two groups.

However, patients within the CoV2 group presented at T1 with slightly lower Hb values and higher ERI levels.

### 3.2. Temporal ERI Patterns in the nonCoV2 and CoV2 Groups

**Figure 1:**
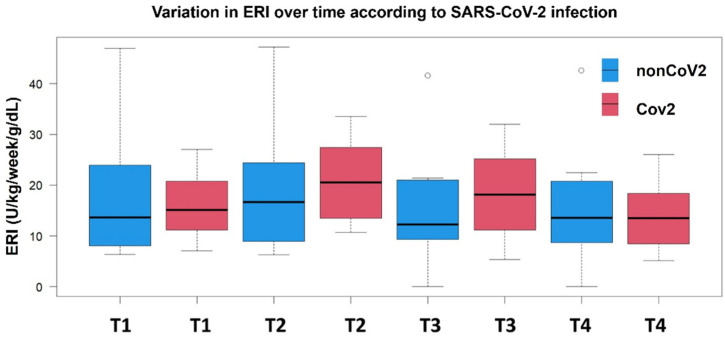
Time-point comparison of ERI levels in patients with and without SARS-CoV-2 infection. Ranges and median ERI values are displayed at each time point from T1 to T4.

The kinetics of the ERI over time and the comparative differences between the two groups are shown in Figure 1. Despite having a nonsignificantly different ERI at T1, the nonCoV2 and CoV2 groups significantly differed in ERI trends over time. Indeed, while both groups exhibited a trend of increased ERI between T1 and T2, the increase was significantly higher among CoV2 patients compared to the nonCoV2 group (*p* < 0.001), with a median ΔERI of +4.65 (1.80–6.25) compared to (−0.27, −1.32–0.21, *p*-value < 0.001) the nonCoV2 patients. Notably, the very wide ranges of ERI measurement for the nonCoV2 group contributed to the slightly negative value of ΔERI_T12_, despite the punctual median ERI value showing a mild positive increase (from 13.6 to 16.6) in this group between T1 and T2.

After the second visit, the ERI slope steeply declined in the CoV2 group (−1.60, −4.35–0.25) compared to the nonCoV2 one (−0.1. −8.92–0.89, *p*-value = 0.03), but among CoV2 patients, it reached baseline-like levels at the fourth visit only. Patients without SARS-CoV-2 infection presented non-significant fluctuations in their ERI across the visits.

The most pronounced between-group difference was observed at T3, where CoV2 patients maintained significantly higher ERI values compared to non-CoV2 ones (difference in the medians = 5.9 U/kg/week/g/dL, *p* = 0.013). By T4, both groups converged to similar ERI levels (13.5, 8.4–18.4 U/kg/week/g/d for CoV2 patients and 13.6, 8.9–19.3 U/kg/week/g/dL for nonCoV2 patients), suggesting a delayed recovery in erythropoietin responsiveness among COVID-19 patients.

This pattern indicates that COVID-19 infection may temporarily impair erythropoietin responsiveness, with maximal effect observed during the intermediate phase of follow-up (T2-T3), followed by eventual recovery to baseline levels.

### 3.3. Factors Associated with Temporal ERI Changes

**Figure 2:**
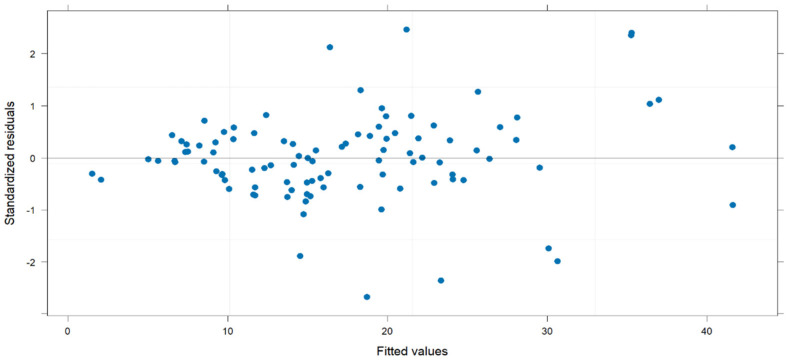
Dispersion plot of standardized residuals against the fitted values.

Linear mixed models were then built to explore the factors associated with ERI trends across the different time points (from T1 to T4). We checked the independence of the random errors using a dispersion plot of the standardized residuals versus the predicted values. As seen in Figure 2, the distribution of residuals was normal and only a few observations were greater than 2 or lower than −2; we observed neither groupings nor trends of observations. Furthermore, the homoscedasticity of variance was respected.

The coefficients and standard errors of factors associated with ERI change between the first (between T1 and T2) and later phases of the follow-up time (between T2 and T4) are reported in Table 2 and Table 3. In brief, between T1 and T2 (Table 2), both groups experienced an overall increase in the ERI over time (β coefficient for time 4.78, 95% CI: 2.41 to 7.15; *p* < 0.001); however, this temporal pattern was not uniform across the groups, as evidenced by the significant time-by-group interaction (β = 4.76 ± 1.21, *p* = 0.02), meaning that CoV2 patients experienced a steeper increase in ERI levels post-SARS-CoV-2 infection. The similar magnitude of the main time effect (4.78) and the interaction term (4.76) suggests a particularly pronounced temporal effect in CoV2 patients compared to nonCoV2 ones. Among other variables, both male sex and hemoglobin values were negatively associated with ERI (β for male sex = −9.51, 95% CI: −16.88 to −2.14; β for hemoglobin, −2.62, 95% CI: −3.68 to −1.56); conversely, age, hypertension, diabetes, and cerebrovascular disease were not significantly associated with ERI changes.

When considering time visits T2–T4 (Table 3), the ERI trend starts to decline over time, but with different rates between the groups; indeed, while the main time effect was no longer significant (β = 1.16 ± 1.03, *p* = 0.27), the time-by-group interaction reversed direction and remained significant (β = −3.65 ± 2.20, *p* = 0.03), indicating a differential recovery pattern between the groups. In particular, CoV2 patients presented a more pronounced but delayed decrease in ERI levels compared to nonCoV2 individuals, possibly reflecting recovery from SARS-CoV-2 acute effects on erythropoiesis. Indeed, the median ERI values within the CoV2 group only mildly decreased between T2 and T3, while the decline was more pronounced at T3.

Bonferroni-corrected post hoc comparisons showed a trend of nonsignificant increase in the ERI in CoV2 patients at T1, and a subsequent significant decrease from T1 to T3 in the CoV2 group only.

## 4. Discussion

In this small-scale retrospective study of 25 patients with advanced CKD treated with HD, we investigated the temporal dynamics of the ERI according to development of SARS-CoV-2 infection, revealing distinct patterns of response over time.

Our analysis identified a biphasic pattern of ESA resistance in patients with SARS-CoV-2 infection. Patients experiencing SARS-CoV-2 infection during the first phase of the follow-up showed an initial and robust increase in ERI values between T1 and T2 (interaction time × group β = 4.76, *p* = 0.02). This pattern reversed during the subsequent period (T2-T4), where CoV2 patients demonstrated a more pronounced decline in ERI values, but with delayed onset (interaction β = −3.65, *p* = 0.03). Notably, male sex emerged as a consistent predictor of lower ERI values across both periods (early: β = −9.51, *p* = 0.02; later: β = −10.60, *p* = 0.01), suggesting gender-specific differences in ESA responsiveness.

These findings highlight the dynamic nature of ESA resistance in patients with SARS-CoV-2 infection undergoing hemodialysis, characterized by an initial phase of increased resistance followed by a delayed but substantial recovery. This temporal evolution has important implications for optimizing ESA dosing strategies in the context of COVID-19 infection.

Several potential mechanisms may contribute to the development of anemia and EPO resistance in response to SARS-CoV-2. First, the inflammatory state elicited by SARS-CoV-2 and characterized by an increased production of proinflammatory cytokines (e.g., IL-1, TNF-α, and IL-6) can lead to enhanced hepcidin production; increased hepcidin levels inhibit intestinal iron absorption and favor iron compartmentalization within macrophages, resulting in low serum iron levels despite normal–high iron stores (functional iron deficiency) [28,29]. As observed in other inflammatory conditions, acute inflammation and low iron availability may also cause the suppression of EPO production and bone marrow hyporesponsiveness [25,26,27]; interestingly, Hanson AL et al. have recently shown how EPO production may vary in response to SARS-CoV-2 infection and the development of COVID-19 [29]. The early postinfectious phase is characterized by a decreased reticulocyte concentration and delayed EPO production, followed by a marked reticulocyte expansion 1–3 months after the infection [29]. This finding was independent of the severity of the disease and was also present in individuals without need of oxygen therapy, thus underlining how the inflammation-related down-regulation of EPO synthesis may overcome the compensatory mechanisms that physiologically upregulate EPO production via hypoxia-inducible factor (HIF) pathways [29]. In this study, patients within the CoV2 group were characterized by EPO hyporesponsiveness, especially during the first months after SARS-CoV-2 infection, with a delayed improvement in ERI values at the last two follow-up visits. EPO hyporesponsiveness may complicate the management of anemia in these patients and lead to suboptimal EPO doses and inadequate treatments, which can be particularly dangerous in patients with advanced CKD. In this regard, a recent cross-sectional study including 96 patients undergoing dialysis showed that increased serum levels of thrombosis-related biomarkers (e.g., plasminogen activator inhibitor-1 and L-type fatty acid binding protein) were associated with an increased risk of EPO hyporesponsiveness [30]; such mediators can be found both in patients with advanced CKD and in those exposed to SARS-CoV-2, two conditions characterized by increased thrombotic risk [31,32]. Furthermore, a study conducted in patients with advanced CKD and cancer has also shown an association between increased C-reactive protein and ESA hyporesponsiveness, thus underlining the role of chronic inflammation in determining ESA hyporesponsiveness in CKD [33]. As SARS-CoV-2 infection triggers the activation of thrombotic cascades and proinflammatory pathways, patients experiencing the infection might consequently be at increased risk of ESA hyporesponsiveness, especially in the early phase post-infection. The endothelial dysfunction associated with COVID-19 adds another layer of complexity to its pathophysiology. The virus’s interaction with Angiotensin-converting enzyme 2 (ACE2) receptors, present on erythroid precursors, may directly impair erythropoiesis [34]. This direct viral effect, combined with the enhanced inflammatory response, creates a particularly challenging environment for maintaining adequate hemoglobin levels in CKD patients. The synergistic effect of these mechanisms often results in increased erythropoietin resistance, a phenomenon that complicates traditional treatment approaches. However, despite these hypotheses, no direct evidence has previously been reported on the impact of SARS-CoV-2 infection on ESA hyporesponsiveness and ERI levels in patients with advanced CKD needing HD. This study highlights a close relationship between infection and resistant anemia, as evidenced by the need to increase the erythropoietin dose in patients with previous infection, even months after diagnosis. These results highlight the importance of considering both temporal changes and group-specific factors in managing anemia in hemodialysis patients, particularly in the context of conditions that might affect erythropoietin responsiveness. Indeed, complete control of anemia has not yet been achieved in high-risk patients, such as those who have had a previous SARS-CoV-2 infection and are suffering the consequences of this disease. In this context, the management of anemia in advanced CKD and COVID-19 might potentially benefit from the administration of HIFs, a possibility that has not yet been fully addressed or explored in the current literature. One of the few available trials evaluating HIFs in renal replacement therapy patients with COVID-19 is the study by Bao et al.; the administration of roxadustat to patients with COVID-19 receiving peritoneal dialysis improved anemia and was well tolerated, especially among individuals who had difficulty taking EPO [35]. Which pharmacological strategy is best for the treatment of resistant anemia in dialysis patients is still debated and the available data are still insufficient. Our preliminary findings represent a starting point for future analyses on larger cohorts to fully elucidate strategies to improve responsiveness to exogenous ESAs in patients with advanced CKD.

Our research has several limitations. First, as it was a single-center study with a small sample size, there was no truly independent external validation cohort; to address this issue, we used one hundred repeated measures, including multiple assessments for each patient. Second, patients with end-stage kidney disease (ESKD) have significant comorbidities and altered physiology that may influence the results. Third, despite all patients being vaccinated during the follow-up time, the absence of a detailed assessment of the number of doses and timing of SARS-CoV-2 vaccination represents a limitation of our study. Finally, our study lacked information about individuals’ residual renal function (RRF) data, as most patients either had no measurable RRF or were unable to provide residual urine for accurate assessment. Despite previous studies having shown no association between RRF and ERI [36], future studies with a larger cohort and comprehensive RRF data collection will be essential to further clarify this relationship. Our study has several notable strengths. First, it is among the few to investigate the ERI in hemodialysis patients with prior SARS-CoV-2 infection, addressing an underexplored but clinically significant area. Second, the use of repeated measures for each patient allows for a robust analysis of ERI trends over time, enhancing the reliability of the findings despite the small sample size. Third, the integration of detailed clinical and laboratory data, including comorbidities and ESA usage, provides a comprehensive understanding of the interplay between SARS-CoV-2 infection, anemia, and erythropoiesis in this high-risk population. Finally, this study highlights the importance of personalized anemia management in hemodialysis patients with prior COVID-19, offering valuable insights for future research and clinical practice. Long-term follow-up of patients in larger cohorts and global data sharing are needed to support these results and the research on this topic.

## 5. Conclusions

Anemia has been associated with poorer clinical outcomes among patients with advanced CKD and COVID-19, including increased mortality rates and longer hospital stays. Our study highlights a strong association between SARS-CoV-2 infection and erythropoietin resistance in patients undergoing HD. This relationship manifests as an initial increase in the erythropoietin resistance index (ERI) following infection, with a delayed recovery phase observed months after diagnosis. These findings underscore the dynamic nature of erythropoietin responsiveness in this vulnerable population and emphasize the importance of tailored anemia management strategies. Further studies are needed to determine the best strategy to prevent COVID-19 in people with kidney disease and improve clinical outcomes such as anemia.

## Figures and Tables

**Table 1:** Descriptive characteristics of the study population and of nonCoV2 and CoV2 groups at the baseline visit (T1).

		All (*n* = 25)	nonCoV2 Group (*n* = 10)	Cov2 Group (*n* = 15)	*p*-Value
Age, mean (SD)		66.5 (14.9)	66.7 (14.4)	66.3 (15.3)	0.913
Male sex, *n* (%)		15 (62.5)	5 (55.6)	10 (66.7)	0.913
Weight, Kg, median (IQR)		74 (67–87.7)	74 (56–86.1)	79 (68.1–95.2)	0.678
Hypertension, *n* (%)		22 (88)	8 (80)	14 (93.3)	0.542
Diabetes mellitus, *n* (%)		7 (28)	1 (10)	6 (40)	0.179
Cancer, *n* (%)		4 (16)	2 (20)	2 (13.3)	0.999
Cerebrovascular disease, *n* (%)		16 (64)	5 (50)	11 (73.3)	0.397
Hb, g/dL, median (IQR)		10.6 (10.0–11.1)	10.4 (9.7–11)	9.9 (9.2–10.9)	0.212
TSAT, %, median (IQR)		15.7 (12.4–21)	15.8 (13.3–25.4)	15.4 (11.2–20.3)	0.650
Ferritin, ng/mL, median (IQR)		138 (68.2–328)	153 (59.4–303)	138 (101–191)	0.244
Transferrin, mg/dL, median (IQR)		197 (174–223)	201 (174–220.7)	195 (163–228)	0.218
ERI, median (IQR)		14.7 (10.7–22.5)	13.6 (8.9–23.5)	15.1 (11.1–20.8)	0.978
KT/V, median (IQR)		1.3 (1.2–1.5)	1.3 (1.2–1.4)	1.3 (1.2–1.5)	0.086
Length of dialysis, days, mean (SD)		238.8 (13.4)	238 (17.3)	240 (0.1)	0.375
Cause of ESKD, *n* (%)					0.16
	Diabetic kidney disease	7 (28.0)	2 (20.0)	5 (33.3)	
	Hypertension	5 (20.0)	2 (20.0)	3 (20.0)	
	Glomerulonephritis	4 (16.0)	4 (40.0)	0	
	ADPKD	3 (12.0)	1 (10.0)	2 (13.3)	
	Unknown	5 (20.0)	1 (10.0)	4 (26.7)	
	Congenital anomalies of kidneys and urinary tract	1 (4.0)	0	1 (6.7)	

Notes: ADPKD = autosomal dominant polycystic kidney disease; ERI = erythropoietin resistance index; ESKD = end-stage kidney disease; Hb = hemoglobin; IQR = interquartile range; SD = standard deviation; TSAT = transferrin saturation.

**Table 2:** Linear mixed models exploring factors associated with ERI between the first and the second follow-up visit, with time expressed as a continuous variable.

Predictor Variable	Estimate	Standard Error	*p*-Value
Age	0.03	0.14	0.80
Time	4.78	1.21	<0.001
Male sex	−9.51	3.76	0.02
Hypertension	−4.03	6.23	0.52
Diabetes	−4.19	4.70	0.38
Cerebrovascular disease	2.47	4.22	0.56
Hb	−2.62	0.54	<0.001
Ferritin	−0.02	0.008	0.12
TSAT	−0.20	0.15	0.20
Kt/V	6.96	6.28	0.28
Length of dialysis	0.06	0.15	0.68
Time × Group	4.76	1.21	0.02
Age × Time	−0.014	0.07	0.85
Sex × Time	2.07	2.16	0.35
Hb × Time	−0.41	−0.43	0.58

Notes: Hb = hemoglobin; TSAT = transferrin saturation.

**Table 3:** Linear mixed models exploring factors associated with ERI between the 2nd and 4th follow-up visits, with time expressed as a continuous variable.

Predictor Variable	Estimate	Standard Error	*p*-Value
Age	0.075	9.21	0.15
Time	1.16	1.03	0.27
Male sex	−10.60	−2.78	0.01
Hemoglobin	0.69	1.24	0.58
Time × Group	−3.65	−2.20	0.03
Age × Time	−0.11	0.05	0.05
Sex × Time	2.23	1.29	0.20
Hb × Time	−1.39	−1.60	0.11

Notes: Hb = hemoglobin.

## Data Availability

The data presented in this study are available on request from the corresponding author due to privacy.

## Abbreviations

The following abbreviations are used in this manuscript:

ADPKD	Autosomal dominant polycistic kidney disease
ACE 2	Angiotensin-converting enzyme 2
ATC	Anatomical Therapeutic Chemical
CKD	Chronic kidney disease
EPO	Erythropoietin
ERI	Erythropoietin resistance index
ESAs	Erythropoietin-Stimulating Agents
ESKD	End-stage kidney disease
Hemoglobin	Hb
HIF	Hypoxia-inducible factor
IQR	Interquartile
HD	Hemodialysis
RT PCR	Real-Time PCR
Standard Deviation	SD
SARS-CoV-2	Severe acute respiratory syndrome coronavirus 2
Transferrin Saturation	TSAT
